# Appropriateness of standard cephalometric norms for the assessment of dentofacial characteristics in patients with cleidocranial dysplasia

**DOI:** 10.1259/dmfr.20210015

**Published:** 2021-11-17

**Authors:** Fabio Savoldi, Francesca Del Re, Ingrid Tonni, Min Gu, Domenico Dalessandri, Luca Visconti

**Affiliations:** 1 Orthodontics, Division of Paediatric Dentistry and Orthodontics, Faculty of Dentistry, the University of Hong Kong, Hong Kong, Hong Kong; 2 Orthodontics, Dental School, Department of Medical and Surgical Specialties, Radiological Sciences and Public Health, University of Brescia, Brescia, Italy; 3 Orthodontics, Dental School, Department of Medical and Surgical Specialties, Radiological Sciences and Public Health, University of Brescia, Brescia, Italy; 4 Orthodontics, Division of Paediatric Dentistry and Orthodontics, Faculty of Dentistry, the University of Hong Kong, Hong Kong, Hong Kong; 5 Orthodontics, Dental School, Department of Medical and Surgical Specialties, Radiological Sciences and Public Health, University of Brescia, Brescia, Italy; 6 Orthodontics, Dental School, Department of Medical and Surgical Specialties, Radiological Sciences and Public Health, University of Brescia, Brescia, Italy

**Keywords:** Cephalometry, Craniofacial, Dental, Orthodontics, Syndrome

## Abstract

**Objectives:**

Cleidocranial dysplasia (CCD) is a rare skeletal syndrome affecting craniofacial and dental development. As a consequence, conventional cephalometric landmarks may not be valid for CCD patients, and the appropriateness of norms used for the general population should be critically discussed.

**Methods:**

Five patients 9- to 22-year-old (three females, two males) with CCD were included. Lateral-cephalograms, orthopantomographies, and intra-oral photos were retrospectively analysed. Lateral-cephalograms of 50 normal controls (ten for each CCD patient) matched for age and sex were selected from an online database. Cephalometric measurements of each CCD patients were compared with average values of matched controls using Wilcoxon signed-rank test for paired values (α = 0.05).

**Results:**

In CCD patients, a shortening of the cranial base was present (*ΔSN* = −17.1 mm, *p* = 0.043). Thus, the mandible (*ΔSNPg* = +9.5°, *p* = 0.043) and the maxilla (*ΔSNA* = +11.2°, *p* = 0.043) showed protrusion compared to the cranial base, despite a reduced maxillary (*ΔCo-A* = −15.1 mm, *p* = 0.043) and mandibular (*ΔCo-Gn* = −15.2 mm, *p* = 0.080) length. The mandibular divergence was reduced (*ΔSN/GoGn* = −6.4°, *p* = 0.043), a reduced overbite was present (*ΔOverbite* = −2.9 mm, *p* = 0.043), and the interincisal angle was increased (*ΔInterincisalAngle* = +13.7°, *p* = 0.043), mainly due to retro-inclination of lower incisors.

**Conclusions:**

Standard cephalometric norms for the assessment of horizontal jaw position may not be applicable to CCD patients because of a reduced anterior cranial base length compared to normal subjects. Vertical relationships may not be affected, and mandibular hypodivergency was confirmed.

## Introduction

Cleidocranial dysplasia (CCD) is a rare genetic syndrome with an estimated prevalence of 1:1.000.000 and characterised by autosomal dominant inheritance.^1^ It is caused by mutations affecting the core-binding factor subunit alpha-1 (Cbfa1)^2^ on the chromosome 6p21.^3^ Involved in the differentiation of osteoblasts,^4^ Cbfa1 is part of the fibroblast growth factor and bone morphogenetic protein pathways in the development of teeth and bones.^5^ Cbfa1 is also a major regulator of chondrocyte differentiation,^6^ related to endochondral formation of long bones and vertebrae.

Commonly, the diagnosis of CCD is clinical, and hypoplastic clavicles, open fontanelles, and supernumerary teeth constitute a characteristic triad.^1^ In patients with atypical characteristics, molecular analysis can be used for differential diagnosis,^7^ as variable loss of function of Cbfa1 may give rise to a clinical variability ranging from isolated primary dental anomalies to classic CCD.^8^

Craniofacial abnormalities are expressed in over 80% of the cases,^9^ including skeletal class III with mandibular prognathism,^10–12^ and a short anterior cranial base.^13,14^ Furthermore, CCD patients often presents a broad forehead, a depressed nasal bridge, a delayed closure of fontanelles and sutures, reduced paranasal sinuses and even missing parietal and nasal bones.^1,15^ Dental signs are expressed in over 90% of patients,^9^ including the retention of the deciduous dentition and the presence of supernumerary teeth,^10,11^ which compromise normal dental eruption. Such anomalies contribute to crowding and malocclusion often including open-bite^11^ and cross-bite.^10,11^

Although previous case-reports have described cephalometric characteristics of patients with CCD^10–12,14^ and reviews have summarised common craniofacial features,^1,9^ the literature is lacking of controlled studies. In fact, it seems that only one previous work compared the cephalometric characteristics of CCD patients with normal subjects, concluding that affected individuals have relatively normal jaw proportions in relation to the cranial base.^16^ However, CCD patients usually present a limited growth of the cranial base,^13^ and it is important to critically analyse the appropriateness of using standard cephalometric norms for these patients. In fact, despite maxillary hypoplasia has been commonly described in CCD patients,^1,9,14^ the reported cephalometric values have been contradictory, showing SNA angles close to 90°.^10,11^

The objective of the present study was to critically investigate the application of conventional cephalometric analysis in CCD patients, to properly understand craniofacial alterations in these subjects.

## Methods and materials

### Subjects

Lateral-cephalograms, orthopantomographies, and intra-oral photos of Caucasian patients affected by CCD from the Dental School of the hospital Spedali Civili di Brescia were retrospectively analysed. Five patients, three females (9-, 13- and 22-years-old) and two males (14- and 16-years-old), were included in the study. In addition, ten lateral-cephalograms matched for sex and age (same year) were selected as controls for each patient with CCD, for a total of 50 controls. Controls were subjects with Angle class I occlusion, which were obtained from the online database of the Craniofacial Growth Legacy Collection.^17^ The study design was modified from Kreiborg et al., which was structured for the comparison of a limited number of rare syndromic cases with a larger control group.^13^ The study was approved by the internal review board of the hospital Spedali Civili di Brescia (Approval number: SINDCRAN NP2882).

### Analysis of lateral radiographs

**Figure 1.**
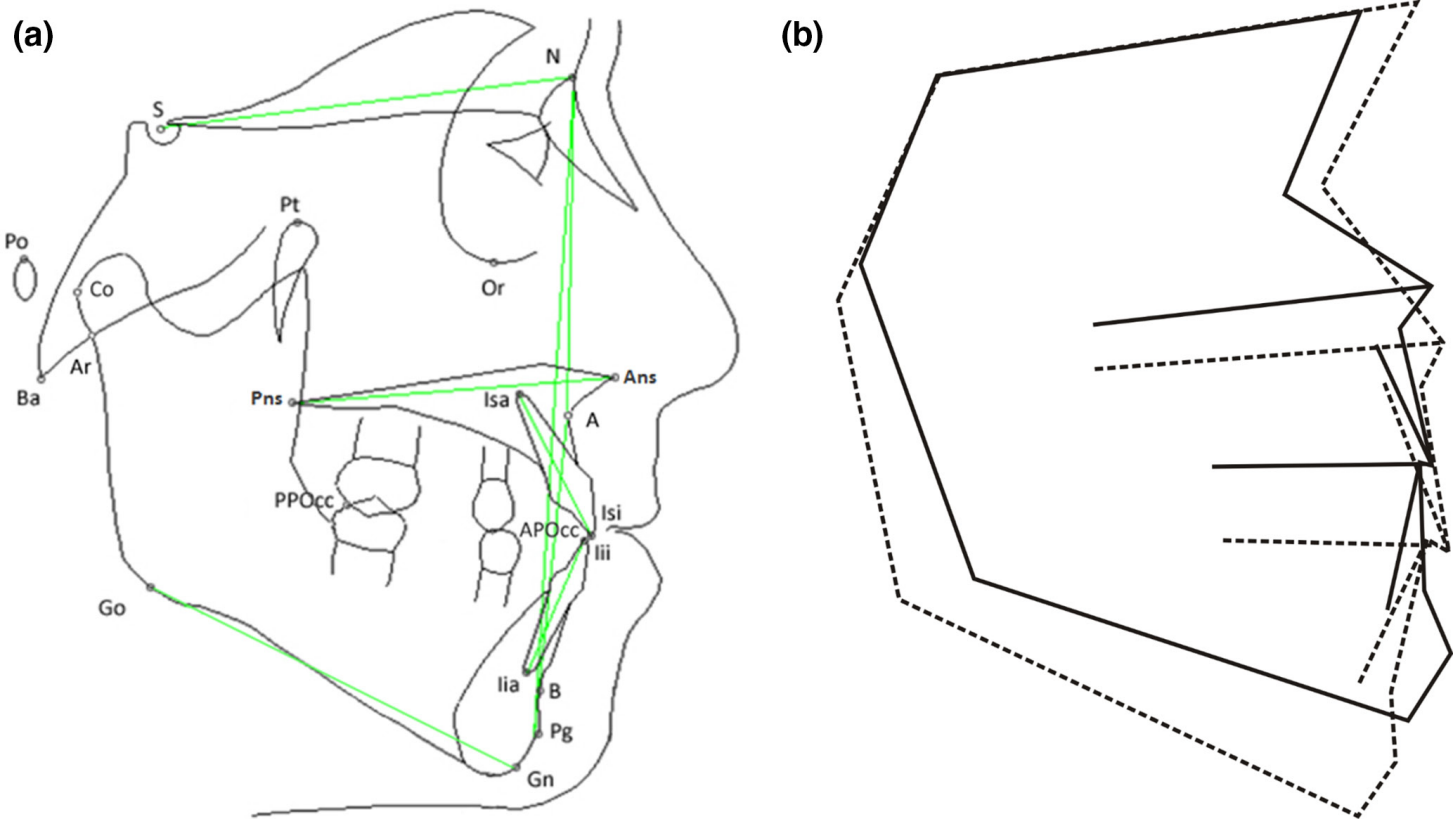
Example of cephalometric tracing showing reference points and planes used for the analysis of the lateral cephalograms (**A**). Superimposition of the average cephalometric tracing of CCD patients (continuous line) and the average tracing of controls (dashed line) (**B**) obtained with cephalometric software (WinCeph V.11, Japan).

The cephalometric analysis was performed using a computer software (OpenCeph 3.3.0, developed by Dr Bruno Oliva). No correction was applied for the X-ray magnification. Horizontal skeletal measurements, vertical skeletal measurements, dento-skeletal measurements, and dento-dental measurements were recorded according to the European Board of Orthodontics guidelines,^18^ with additional parameters from the analysis of Jacobson,^19^ McNamara,^20^ and Jarabak^21^ (Table 1 and Figure 1A).

**Table 1.** Identification of anatomical points adopted in the cephalometric analysis

Point	Name	Description
A	A point	The deepest point in the concavity of the anterior maxilla between the anterior nasal spine and the alveolar crest
APOcc	Anterior point of occlusion	The midpoint of the overbite of the central incisors
Ar	Articular point	The point of intersection of the posterior margin of the ascending mandibular ramus and the outer margin of the cranial base
B	B point	The deepest point in the concavity of the anterior mandible between the alveolar crest and the pogonion
Ba	Basion	The most inferior point of the anterior margin of the foramen magnum
Co	Condilion	The most posterior point of the head of the mandibular condyle
Gn	Gnathion	The most anterior and inferior point of the bony chin
Go	Gonion	The point of intersection of the mandibular plane with the line tangent to the distal margin of the mandibular ramus
Lia	Lower incisor apex	The apex of the root of the lower central incisor
Lii	Lower incisor incisal	The incisal margin of the lower central incisor
Uia	Upper incisor apex	The apex of the root of the upper central incisor
Uii	Upper incisor incisal	The incisal margin of the upper central incisor
N	Nasion	The most anterior point of the fronto-nasal suture
Or	Orbitale	The most inferior point of the inferior bony margin of the orbit
Pg	Pogonion	The most anterior point of the anterior margin of the bony chin
Po	Porion	The most superior point of the external auditory meatus
PPOcc	Posterior point of occlusion	The most distal contact point of the first molars
Pt	Pterigoideo	The most superior point of the pterigo-maxillary fissure
S	Saddle	The point at the centre of the *sella turcica*
Ans	Anterior nasal spine	The anterior extremity of the bony palate
Pns	Posterior nasal spine	The posterior extremity of the bony palate

**Figure 2.**
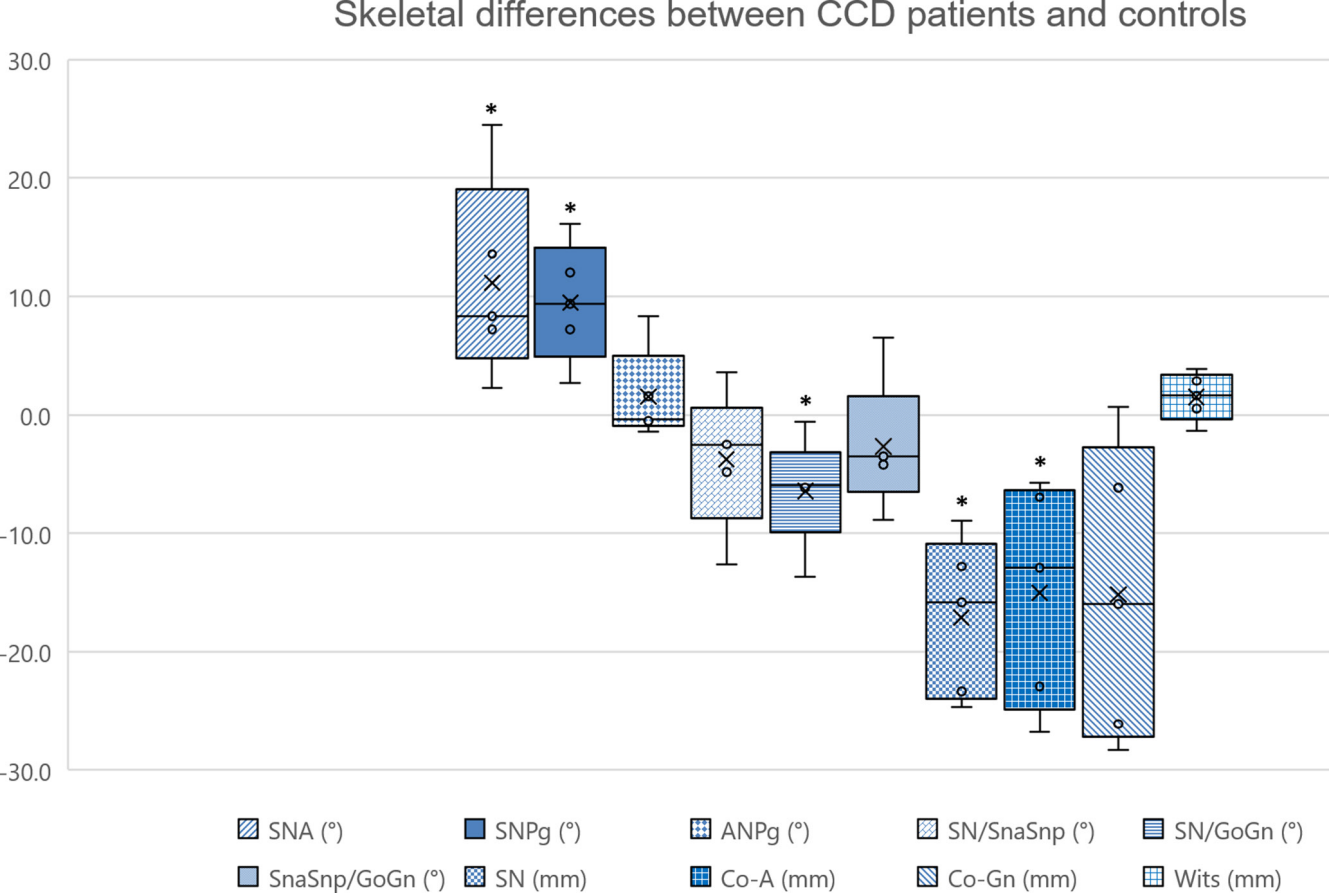
Skeletal differences between CCD patients and controls. The asterisks (*) indicate a statistically significant difference (*p* < 0.05), and bars indicate the interquartile range (IQR).

**Figure 3.**
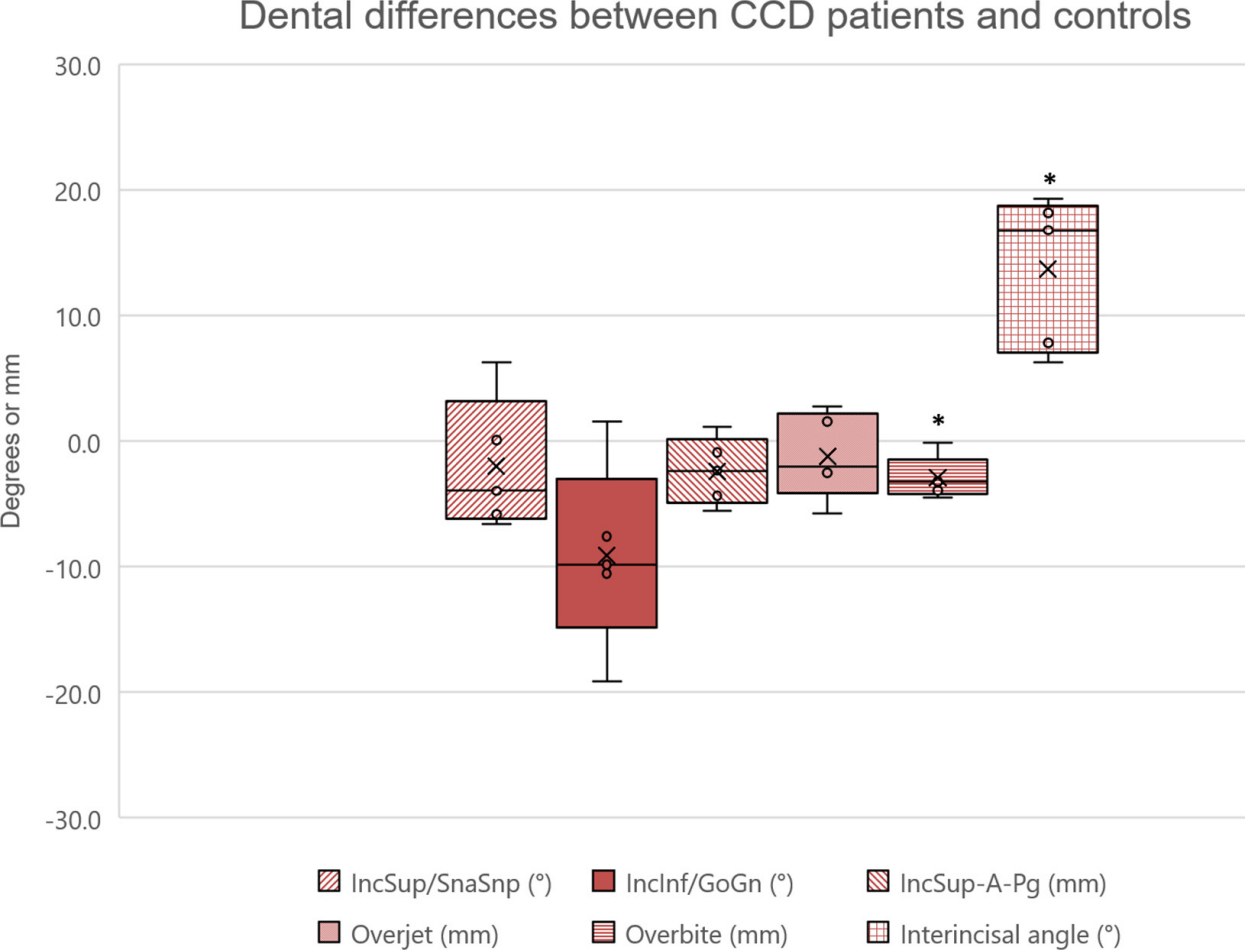
Dental differences between CCD patients and controls. The asterisks (*) indicate a statistically significant difference (*p* < 0.05), and bars indicate the interquartile range (IQR).

### Statistical analysis

After an initial calibration on five lateral cephalograms, measurements were taken by one assessor (F.D.R.), who repeated the measurements after a wash-out period of about one month. The single measure intraclass correlation coefficient (ICC) for absolute agreement was used to calculate the intra-assessor agreement.^22^ ICC was considered “poor” if <0.5, “fair” from 0.5 to 0.7, “good” from 0.7 to 0.8, and “excellent” if >0.8.^23^ The method error was estimated with Dahlberg’s formula.^24^ The average between the two repeated measurements was calculated and used for analysis. Results were reported as mean and standard deviation (SD). The data of each CCD patient were compared with the average value of 10 matched controls. Non-parametric tests were adopted, and the two groups were compared with the Wilcoxon signed-rank test for paired values. Data analysis was performed with statistical software (SPSS^©^ V23.0, IBM, US) at significance level α = 0.05. Orthopantomographies and intra-oral photos of CCD patients were used for further qualitative description of dental and facial characteristics.

## Results

The intra-assessor agreement was generally lower for measurements involving the incisors and higher for the others, ranging between 0.628 (*IncInf/GoGn*) and 0.908 (*SNA*) for angular measurements, and between 0.428 (*Overbite*) and 0.995 (*SN*) for linear measurements. The method error ranged between 1.6° (*ANPg*) and 4.8° (*InterincisalAngle*) for angular measurements, and between 0.4 mm (*Wits*) and 2.0 mm (*Co-Gn*) for linear measurements.

### Skeletal characteristics

Even though in CCD patients the maxillary length was reduced (*ΔCo-A* = −15.1 mm, *p* = 0.043), the maxilla was protruded respect to the anterior cranial base (*SNA* = 92.8°), showing a significant difference compared to controls (*ΔSNA* =+11.2°, *p* = 0.043). Similarly, although in CCD patients the mandible was shorter (*ΔCo-Gn* = −15.2 mm), the mandible was protruded respect to the anterior cranial base (*SNPg* = 91.1°), showing a significant difference compared to controls (*ΔSNPg* =+9.5°, *p* = 0.043). However, the cranial base was shorter in CCD patients compared to controls (*ΔSN* = −17.1 mm, *p* = 0.043). Overall, both measurements representing the antero-posterior jaw discrepancy did not show significant differences between CCD patients and controls (*ΔANPg* =+1.5°, and *ΔWits* =+1.5 mm). In addition, the mandible of CCD patients was hypodivergent compared to controls (*ΔSN/GoGn* = −6.4°, *p* = 0.043) (Figures 1A and 2).

**Table 2.** Summary of the dento-skeletal parameters of CCD patients and controls

			CCD patients							Controls							Difference		
			Case 1	Case 2	Case 3	Case 4	Case 5			CTR 1^a^	CTR 2^a^	CTR 3^a^	CTR 4^a^	CTR 5^a^					
Measurement	Unit	Age (years)	9	13	14	16	22	Mean	SD	9	13	14	16	22	Mean	SD	Mean	SD	*p*-value^b^
SNA	°		102.5	84.0	90.8	95.4	91.2	92.8	6.8	78.0	81.7	83.6	81.8	82.8	81.6	2.1	11.2	4.7	**0.043**
SNPg	°		93.7	84.3	90.6	94.8	92.1	91.1	4.1	77.5	81.7	83.3	82.8	82.7	81.6	2.4	9.5	1.8	**0.043**
ANPg	°		8.9	−0.3	0.2	0.6	−0.9	1.7	4.0	0.5	0.1	0.7	−1.0	0.5	0.2	0.7	1.5	3.3	0.686
SN/SnaSnp	°		−5.3	8.1	−0.2	2.0	0.7	1.1	4.8	7.4	4.5	2.3	4.5	5.5	4.8	1.9	−3.8	2.9	0.223
SN/GoGn	°		26.8	31.0	23.7	23.7	16.0	24.2	5.5	32.9	31.6	29.5	29.6	29.6	30.6	1.5	−6.4	4.0	**0.043**
SnaSnp/GoGn	°		32.0	22.9	23.9	21.6	15.3	23.1	6.0	25.5	27.1	27.2	25.1	24.2	25.8	1.3	−2.7	4.7	0.345
SN	mm		57.0	60.0	66.0	58.0	59.0	60.0	3.5	81.7	72.8	74.9	81.4	74.8	77.1	4.1	−17.1	−0.6	**0.043**
Co-A	mm		70.8	72.5	78.3	71.5	81.9	75.0	4.9	93.7	85.4	84.0	98.3	88.9	90.1	5.9	−15.1	−1.1	**0.043**
Co-Gn	mm		98.7	99.9	112.6	108.7	113.1	106.6	6.9	124.8	115.8	111.9	137.0	119.3	121.7	9.7	−15.2	−2.8	0.080
Wits	mm		−0.5	−2.5	4.5	−1.5	2.0	0.4	2.8	−1.0	−1.1	0.6	−3.1	−0.9	−1.1	1.3	1.5	1.5	0.138
IncSup/SnaSnp	°		104.3	106.9	102.3	121.4	111.7	109.3	7.6	110.8	110.9	108.1	115.1	111.6	111.3	2.5	−2.0	5.1	0.500
IncInf/GoGn	°		72.9	78.3	85.5	82.5	92.9	82.4	7.5	92.0	88.1	93.1	93.0	91.4	91.5	2.0	−9.1	5.5	0.080
IncSup-A-Pg	mm		−2.0	2.0	1.2	−1.0	−1.1	−0.2	1.7	2.4	0.9	2.1	4.5	1.3	2.2	1.4	−2.4	0.3	0.138
Overjet	mm		1.8	−1.9	0.6	5.0	5.6	2.2	3.1	4.3	3.9	2.6	3.4	2.8	3.4	0.7	−1.2	2.4	0.500
Overbite	mm		−0.6	0.1	−2.0	2.1	−1.6	−0.4	1.6	2.3	3.3	2.6	2.2	2.3	2.5	0.4	−2.9	1.2	**0.043**
Interincisal angle	°		150.9	152.0	148.4	134.7	140.2	145.2	7.5	131.6	133.8	131.6	126.8	133.9	131.6	2.9	13.7	4.6	**0.043**

SD = standard deviation; CTR = control

Significant *p*-values are reported in bold

a Mean of 10 matched controls from the American Association of Orthodontists Foundation

b =Wilcoxon signed rank test

### Dental characteristics

In CCD patients, the interincisal angle was significantly higher compared to controls (*ΔInterincisalAngle* =+13.7°, *p* = 0.043), mainly due to retro-inclination of the lower incisors (*ΔIncInf/GoGn* = −9.1°). An anterior open-bite (*overbite* = −0.4 mm) was also present in CCD patients compared to controls (Table 2, Figures 1B and 3), as confirmed by the intra-oral photos (Supplementary Material 1). All patients presented one or multiple retained deciduous teeth. One patient showed impacted upper central and lateral incisors, and one patient an impacted upper second premolar. Furthermore, one patient presented supernumerary upper central incisors (Table 3).

Supplementary Material 1.

**Table 3.** Dental formula and dental history of the analysed CCD cases. Teeth were marked according to FDI World Dental Federation notation

	Age	18	17	16	15	14	13	12	11	21	22	23	24	25	26	27	28	38	37	36	35	34	33	32	31	41	42	43	44	45	46	47	48
Case 1	9	NE	NE	✓	55	54	53	52	51	61	62	63	64	65	✓	NE	NE	NE	NE	✓	75	74	73	72	✓	✓	82	83	84	85	✓	NE	NE
Case 2	13	NE	✓	✓	55	54	53	52	51+1	61+1	62	63	✓	65	✓	✓	NE	NE	✓	✓	75	74	73	✓	✓	✓	82	83	84	85	✓	✓	NE
Case 3	14	NE	✓	✓	55	54	53	IM	IM	✓	IM	63	✓	✓	✓	✓	NE	NE	✓	✓	✓	✓	✓	✓	✓	✓	✓	43	✓	✓	✓	NE	NE
Case 4	16	NE	NE	✓	✓	✓	✓	✓	✓	✓	✓	✓	✓	✓	✓	EX	NE	NE	✓	✓	✓	✓	73	✓	✓	✓	✓	EX	✓	✓	✓	NE	NE
Case 5	22	NE	✓	✓	IM	✓	53	✓	✓	✓	✓	✓	✓	✓	✓	✓	NE	NE	✓	✓	✓	✓	73	✓	✓	✓	✓	83	✓	✓	✓	✓	NE

EX = extraction; NE = not erupted; IM = impacted;+1=supernumerary

### Other characteristics

In four subjects, the nasal bones were difficult to identify on cephalometric radiographs, probably due to their underdevelopment or absence. No other evident abnormality of craniofacial bones was noticed. Although the frontal sinus was not recognisable on cephalometric radiographs, the maxillary sinuses appeared normally developed on cephalometric and panoramic radiographs. No palatoschisis was noticed from radiographs or intra-oral photos (Supplementary Material 1).

## Discussion

### Skeletal characteristics

The present study showed a mean *SNA* angle of 92.8° in CCD patients, which was significantly higher compared to controls (+11.2°), indicating maxillary protrusion with respect to a normal value of about 81.0°.^25^ Accordingly, previous studies analysing CCD patients reported *SNA* values ranging from 87.4° to 97.0°,^10–12^ confirming a forward position of the maxilla with respect to the anterior cranial base. Since the *SNA* angle may not discriminate the contribution of maxillary size to the overall maxillary position, the maxillary length was also measured. Perhaps surprisingly, and in disagreement with a high *SNA* angle, a significantly shorter maxilla was shown in CCD patients compared to controls (*ΔCo-A* = −15.1 mm), in agreement with the published literature.^1,9–11,14^

Regarding the mandible, the present study showed a mean *SNPg* value of 91.1°, which was significantly higher than the controls (+9.5°). Accordingly, previous studies reported CCD patients to present SNB values ranging from 87.4° to 97.0°,^10–12^ supporting the presence of the prognathic mandible described in the literature.^1,14,16^ However, the *Co-Gn* values did not show significant differences respect to controls and suggested a norma- or even hypoplasti-mandible instead. Therefore, the clinically evident class III tendency may be more attributable to the marked midface hypoplasia rather than mandibular hyperplasia.^14^ Yet, the *ANPg* value (1.7°) did not show significant differences compared to controls and previous studies in CCD patients reported ANB values ranging between 0.0° and 3.0°,^10,12^ which are compatible with a norm of about 3.0°.^25^ In addition, the *Wits* value (1.5 mm) did not show significant differences compared to controls. These two aspects could be explained by the fact that the ANB angle increases if Nasion is positioned more posteriorly, while the *Wits* appraisal increases if the occlusal plane is rotated counter clockwise.^26^ In fact, the anterior cranial base is short in CCD patients,^1,11,13,14^ leading to a posterior position of Nasion. Previous authors reported an average anterior cranial base length in CCD adults of 63.1 mm for females and 70.3 mm for males, compared to 70.4 mm and 73.4 mm in normal controls, respectively.^13^ Other authors showed cases of CCD patients with anterior cranial base as short as 57.6 mm at 12-year-old, and 58.4 mm at 14-year-old.^11^ In the present study, the average length of the anterior cranial base was 60.0 mm, with a shortening of 17.1 mm compared to controls. Since the whole mid-face of CCD patients is usually poorly developed and further compromised by small or absent nasal bones,^1^ the anterior cranial base may not be a suitable reference for the assessment of antero-posterior jaw relationships in these patients. As reported by Jarvinen for CCD,^12^ and also supported by Jacobson for skeletal class III,^27^ the ANB angle can be erroneous in presence of facial prognathism and “*the impression of normal or nearly normal sagittal relation between the jaws [in CCD patients] is regarded as misleading*”.^12^ Similarly, *ANPg* values may be increased due to a shortening of the anterior cranial base. Accordingly, Binder demonstrated a change of 2.5° in the ANB angle for a 5.0 mm horizontal displacement of Nasion,^28^ and Mills suggested a correction of the ANB angle of −0.5° for each degree of deviation of the SNA angle above the upper normative value (81°+3°).^29^ Alternative cephalometric methods that use the forehead as a reference^30^ may be not advisable as well, as the forehead position is also likely to be affected by CCD. Instead, establishing a coordinate system originating from Sella may offer advantages, such as in patients affected by craniosynostosis.^31^

Beside the controversial assessment of antero-posterior relationships, a mandibular hypodivergence in CCD patients has been consistently reported in the literature with *SN/Go-Gn* values of 23.4°^10^ and 21.1°,^16^ compared to a norm of about 29.0°.^25^ The present study confirmed a counter clockwise mandibular rotation, showing an average *SN/GoGn* angle of 24.2° that was significantly reduced compared to controls (−6.4°). Such forward rotation of the mandible might be caused by a reduced vertical development of the midface, which may be related to hypoplasia of facial bones and underdevelopment of paranasal sinuses.^1^ In fact, nasal bones were difficult to identify on cephalometric radiographs of most patients, and a depressed nasal bridge was present. In addition, the vertical facial growth may be decreased due to a reduced alveolar bone development related to lack of eruption of permanent teeth,^16^ eventually contributing to a brachifacial phenotype. Thus, clinicians may consider spontaneous or orthodontic-guided eruption, and prosthodontic rehabilitation for increasing the lower anterior facial height and the mandibular divergence.^11^

### Dental characteristics

In the present study, the interincisal angle (145.2°) was significantly larger in CCD patients compared to controls (+13.7°). The marked lingual inclination of lower incisors (82.4°) confirmed the skeletal class III tendency, as noticed by previous authors reporting *IncInf/GoGn* values from 65.5° to 76.0°.^12^ In addition, the anterior open-bite reported in previous studies^10,11^ was confirmed by the present findings (*ΔOverbite* = −2.9 mm). Given the hypodivergent mandibular growth and the brachicephalic skeletal pattern, the open-bite should be considered of dental origin rather than skeletal.

With regard to dental anomalies in CCD patients, hyperdontia is among the most reported in the literature,^32,33^ and the presence of supernumeraries may be due to incomplete resorption of the dental lamina.^34^ Supernumerary elements can be either uniformly or chaotically located in the jaws, with the upper and lower dentition similarly affected.^35^ The altered eruption seems to be consequent to the obstruction created by the lack of resorption of the deciduous teeth roots and respective alveolar bone, which is caused by hyperdontia.^36^ Accordingly, the CCD subjects evaluated in the present study exhibited retention of deciduous elements and supernumerary permanent teeth, especially in the frontal region. None of the patients presented agenesis, which have been reported only in few cases.^11^

### Limitations

The main limitation of the present work was the sample size, which is a common issue in studies analysing CCD patients, given the rare incidence of this syndrome. Despite the wide age-range analysed, the comparison with matched controls ensured a fair assessment accounting for growth differences. However, due to the retrospective nature of the study and the use of a sample from an online database, the acquisition methods of the lateral-cephalograms may have varied. Furthermore, the incisal position was difficult to assess in CCD patients because of the disodontiasis that affected the anterior region, as confirmed by the poor intra-assessor agreement for the overbite and the large method error for the interincisal angle. In addition, some patients were in primary while others in permanent dentition, and one patient received fixed multibracket treatment, which may have influenced the incisal position as well. Thus, cephalometric data of the incisal area should be considered with caution. Additional studies are needed to understand the underlying growth mechanism related to the described craniofacial characteristics, including radiological assessments of the sutural development.^37^ Furthermore, cephalometric assessments may investigate the effects of the craniofacial morphology of CCD patients on the upper airway.^38^

## Conclusions

In CCD patients, jaws resulted protruded with respect to the anterior cranial base despite the presence of maxillary hypoplasia. However, the anterior cranial base was shorter in CCD patients compared to controls, and normative values used for the diagnosis of antero-posterior jaws position in normal subjects may not be applicable.

In CCD patients, cephalometric analyses using the anterior cranial base as a reference should be critically re-interpreted to avoid misleading diagnosis. Nevertheless, conventional cephalometric analysis may still be valuable for assessing treatment changes.

Supero-inferior values were less affected by the antero-posterior length of the anterior cranial base, and the hypodivergent mandible described in the literature was confirmed.

Further studies are necessary to confirm the present findings and to understand the respective underlying growth mechanism.

## References

[1] Mundlos S. Cleidocranial dysplasia: clinical and molecular genetics. J Med Genet 1999; 36: 177–82. 10204840 PMC1734317

[2] Mundlos S, Otto F, Mundlos C, Mulliken JB, Aylsworth AS, Albright S, et al. Mutations involving the transcription factor Cbfa1 cause cleidocranial dysplasia. Cell 1997; 89: 773–9. doi: 10.1016/S0092-8674(00)80260-3 9182765

[3] Mundlos S, Mulliken JB, Abramson DL, Warman ML, Knoll JH, Olsen BR. Genetic mapping of cleidocranial dysplasia and evidence of a microdeletion in one family. Hum Mol Genet 1995; 4: 71–5. doi: 10.1093/hmg/4.1.71 7711736

[4] Ducy P, Zhang R, Geoffroy V, Ridall AL, Karsenty G. Osf2/Cbfa1: a transcriptional activator of osteoblast differentiation. Cell 1997; 89: 747–54. doi: 10.1016/S0092-8674(00)80257-3 9182762

[5] James MJ, Järvinen E, Wang X-P, Thesleff I. Different roles of Runx2 during early neural crest-derived bone and tooth development. J Bone Miner Res 2006; 21: 1034–44. doi: 10.1359/jbmr.060413 16813524

[6] Kim IS, Otto F, Zabel B, Mundlos S. Regulation of chondrocyte differentiation by Cbfa1. Mech Dev 1999; 80: 159–70. doi: 10.1016/S0925-4773(98)00210-X 10072783

[7] Hall BD. Syndromes and situations associated with congenital clavicular hypoplasia or agenesis. In: Papadatos C. J, Bartsocas C. S, eds.. New York: Alan R. Liss: Skeletal dysplasias; 1982. pp. 279–88. 7163272

[8] Zhou G, Chen Y, Zhou L, Thirunavukkarasu K, Hecht J, Chitayat D, et al. Cbfa1 mutation analysis and functional correlation with phenotypic variability in cleidocranial dysplasia. Hum Mol Genet 1999; 8: 2311–6. doi: 10.1093/hmg/8.12.2311 10545612

[9] Golan I, Baumert U, Hrala BP, Müssig D. Dentomaxillofacial variability of cleidocranial dysplasia: clinicoradiological presentation and systematic review. Dentomaxillofac Radiol 2003; 32: 347–54. doi: 10.1259/dmfr/63490079 15070835

[10] Farronato G, Maspero C, Farronato D, Gioventù S. Orthodontic treatment in a patient with cleidocranial dysostosis. Angle Orthod 2009; 79: 178–85. doi: 10.2319/111307-393.1 19123713

[11] Park TKN, Vargervik K, Oberoi S. Orthodontic and surgical management of cleidocranial dysplasia. Korean J Orthod 2013; 43: 248–60. doi: 10.4041/kjod.2013.43.5.248 24228240 PMC3822065

[12] Järvinen S. Cephalometric findings in three cases of cleidocranial dysostosis. Am J Orthod 1981; 79: 184–91. doi: 10.1016/0002-9416(81)90316-X 6937141

[13] Kreiborg S, Jensen BL, Björk A, Skieller V. Abnormalities of the cranial base in cleidocranial dysostosis. Am J Orthod 1981; 79: 549–57. doi: 10.1016/S0002-9416(81)90465-6 6940449

[14] Lee C, Jung H-S, Baek J-A, Leem DH, Ko S-O, . Manifestation and treatment in a cleidocranial dysplasia patient with a Runx2 (T420I) mutation. Maxillofac Plast Reconstr Surg 2015; 37: 41. doi: 10.1186/s40902-015-0042-0 26594640 PMC4643116

[15] Savoldi F, Tsoi JKH, Paganelli C, Matinlinna JP. The biomechanical properties of human craniofacial sutures and relevant variables in sutural distraction osteogenesis: a critical review. Tissue Eng Part B Rev 2018; 24: 25–36. doi: 10.1089/ten.teb.2017.0116 28610544

[16] Ishii K, Nielsen IL, Vargervik K. Characteristics of jaw growth in cleidocranial dysplasia. Cleft Palate Craniofac J 1998; 35: 161–6. doi: 10.1597/1545-1569_1998_035_0161_cojgic_2.3.co_2 9527313

[17] AAOF.Craniofacial Growth Legacy Collection. American Association of Orthodontists Foundation: Craniofacial Research Instrumentation Laboratory, Department of Orthodontics, University of the Pacific; 2017.

[18] Sandler PJ, Duterloo HS. European board of Orthodontists: a professional challenge. J Orthod 2003; 30: 59–71. doi: 10.1093/ortho/30.1.59 12644609

[19] Jacobson A. The "Wits" appraisal of jaw disharmony. Am J Orthod 1975; 67: 125–38. doi: 10.1016/0002-9416(75)90065-2 1054214

[20] McNamara JA. A method of cephalometric evaluation. Am J Orthod 1984; 86: 449–69. doi: 10.1016/S0002-9416(84)90352-X 6594933

[21] Jarabak JR, Fizzel JA. In: Technique and treatment with lightwire appliances. 2nd ed. St. Louis (MO); 1972.

[22] Shrout PE, Fleiss JL. Intraclass correlations: uses in assessing rater reliability. Psychol Bull 1979; 86: 420–8. doi: 10.1037/0033-2909.86.2.420 18839484

[23] Blacker D. Psychiatric Rating Scales. In: Sadock B. J, Sadock V, eds.Comprehensive Textbook of Psychiatry. 8th ed. Philadelphia: Lippincott Williams & Wilkins; 2005. pp. 929–55.

[24] Dahlberg G. Statistical methods for medical and biological students. Br Med J 1940; 2: 358–9.

[25] Reyes BC, Baccetti T, McNamara JA. An estimate of craniofacial growth in class III malocclusion. Angle Orthod 2006; 76: 577–84. doi: 10.1043/0003-3219(2006)076[0577:AEOCGI]2.0.CO;2 16808562

[26] Jacobson A. Update on the Wits appraisal. Angle Orthod 1988; 58: 205–19. doi: 10.1043/0003-3219(1988)058<0205:UOTWA>2.0.CO;2 3056122

[27] Jacobson A. Application of the "Wits" appraisal. Am J Orthod 1976; 70: 179–89. doi: 10.1016/S0002-9416(76)90318-3 1066054

[28] Binder RE. The geometry of cephalometrics. J Clin Orthod 1979; 13: 258–63. 296153

[29] Mills JRE. Principles and Practice of Orthodontics. New York: Churchill Livingstone; 1982. pp. 70–85.

[30] Andrews WA. Ap relationship of the maxillary central incisors to the forehead in adult white females. Angle Orthod 2008; 78: 662–9. doi: 10.2319/0003-3219(2008)078[0662:AROTMC]2.0.CO;2 18302465

[31] Gibson TL, Grayson BH, McCarthy JG, Shetye PR. Maxillomandibular and occlusal relationships in preadolescent patients with syndromic craniosynostosis treated by LeFort III distraction osteogenesis: 10-year surgical and phenotypic stability. Am J Orthod Dentofacial Orthop 2019; 156: 779–90. doi: 10.1016/j.ajodo.2018.12.022 31784011

[32] Dalessandri D, Laffranchi L, Tonni I, Zotti F, Piancino MG, Paganelli C, et al. Advantages of cone beam computed tomography (CBCT) in the orthodontic treatment planning of cleidocranial dysplasia patients: a case report. Head Face Med 2011; 7: 6. doi: 10.1186/1746-160X-7-6 21352577 PMC3053235

[33] Symkhampha K, Ahn GS, Huh K-H, Heo M-S, Lee S-S, Kim J-E. Radiographic features of cleidocranial dysplasia on panoramic radiographs. Imaging Sci Dent 2021; 51: 271–8. doi: 10.5624/isd.20201007 34621654 PMC8479436

[34] Roberts T, Stephen L, Beighton P. Cleidocranial dysplasia: a review of the dental, historical, and practical implications with an overview of the South African experience. Oral Surg Oral Med Oral Pathol Oral Radiol 2013; 115: 46–55. doi: 10.1016/j.oooo.2012.07.435 23102800

[35] Yamamoto H, Sakae T, Davies JE. Cleidocranial dysplasia: a light microscope, electron microscope, and crystallographic study. Oral Surg Oral Med Oral Pathol 1989; 68: 195–200. doi: 10.1016/0030-4220(89)90192-8 2780020

[36] Proffit WR, Fields HW, Sarver DM. Contemporary Orthodontics. Missouri: Elsevier 2013;.

[37] Savoldi F, Tsoi JKH, Paganelli C, Matinlinna JP. Sutural morphology in the craniofacial skeleton: a descriptive Microcomputed tomography study in a swine model. Anat Rec 2019; 302: 2156–63. doi: 10.1002/ar.24230 31433566

[38] Savoldi F, Xinyue G, McGrath CP, Yang Y, Chow SC, Tsoi JKH, et al. Reliability of lateral cephalometric radiographs in the assessment of the upper airway in children: *A retrospective study*. Angle Orthod 2020; 90: 47–55. doi: 10.2319/022119-131.1 31403838 PMC8087055

