# Supplementary material for: Appropriateness of standard cephalometric norms for the assessment of dentofacial characteristics in patients with cleidocranial dysplasia
Source: Dentomaxillofac Radiol. 2021 Nov 17;51(3):20210015. doi: 10.1259/dmfr.20210015 (PMC8925878; doi:10.1259/dmfr.20210015)
Supplement: Supplementary Material 1. [file dmfr.20210015.suppl-01.docx]

**APPENDIX**


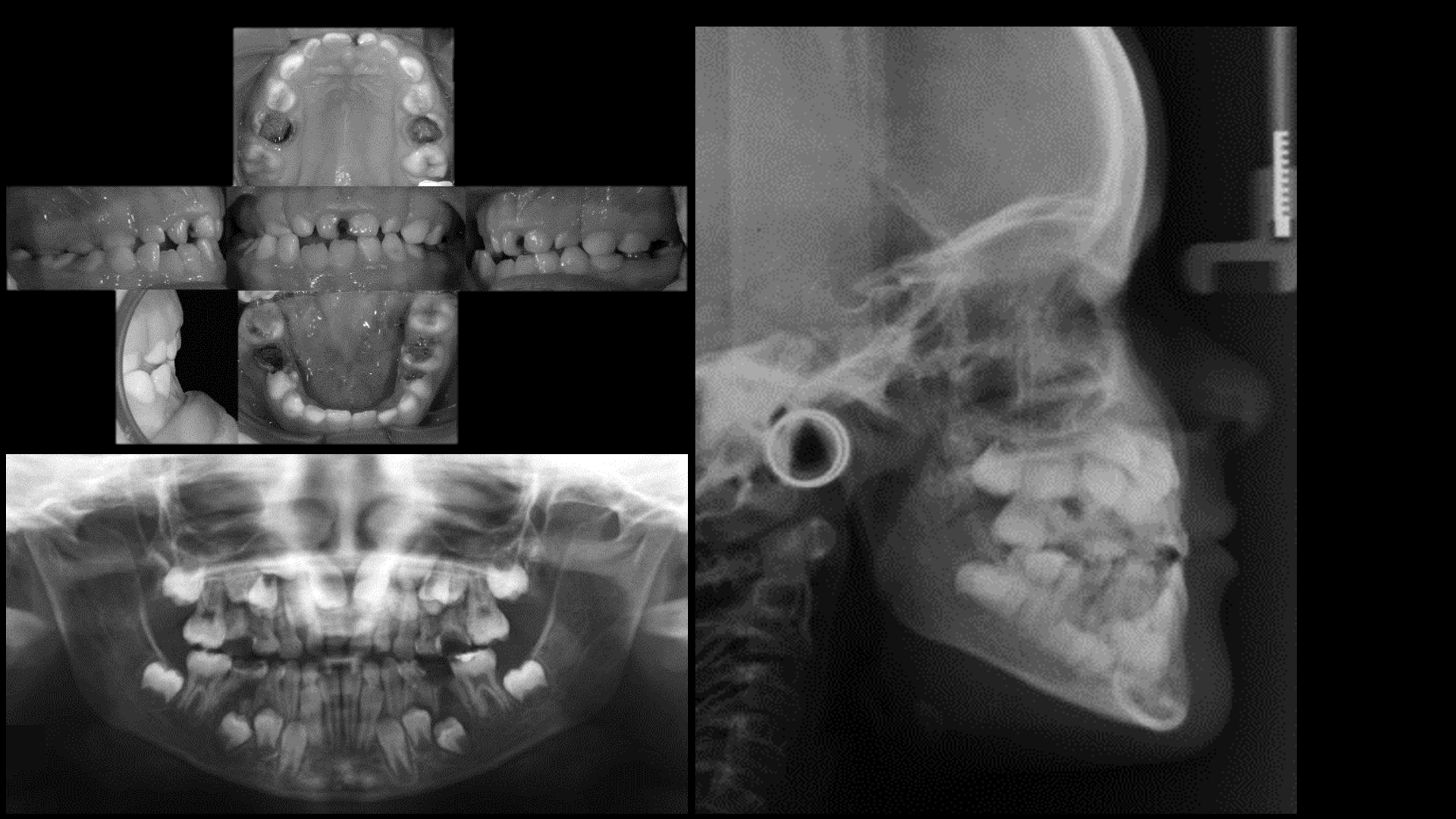


**Appendix figure 1:** CCD case one (nine year-old). Intraoral photos, orthopantomography and lateral cephalogram.


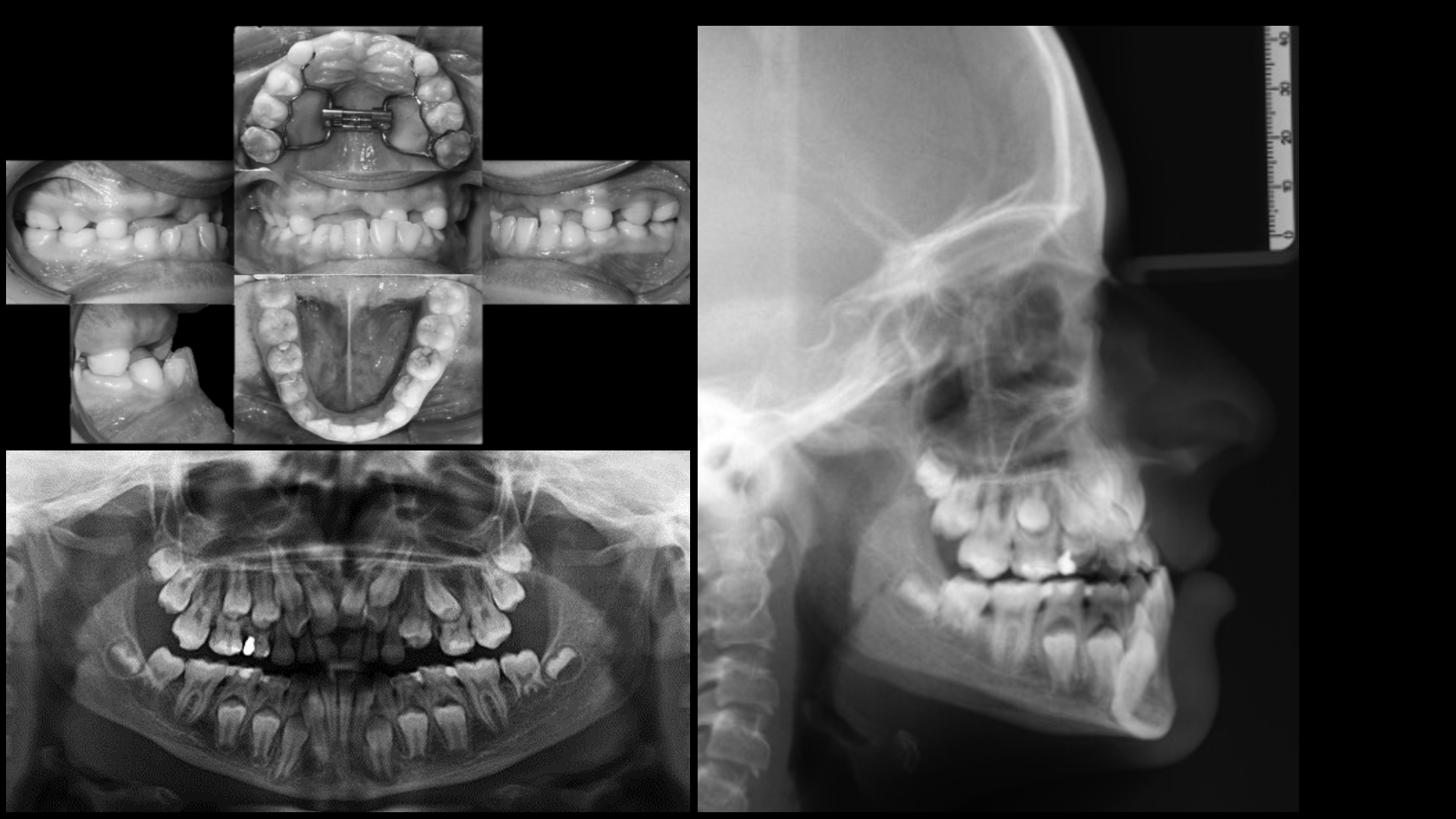


**Appendix figure 2:** CCD case two (thirteen year-old). Intraoral photos, orthopantomography and lateral cephalogram. Rapid maxillary expansion was performed after acquisition of orthopantomography and lateral cephalogram, and it did not influence the radiographic findings.


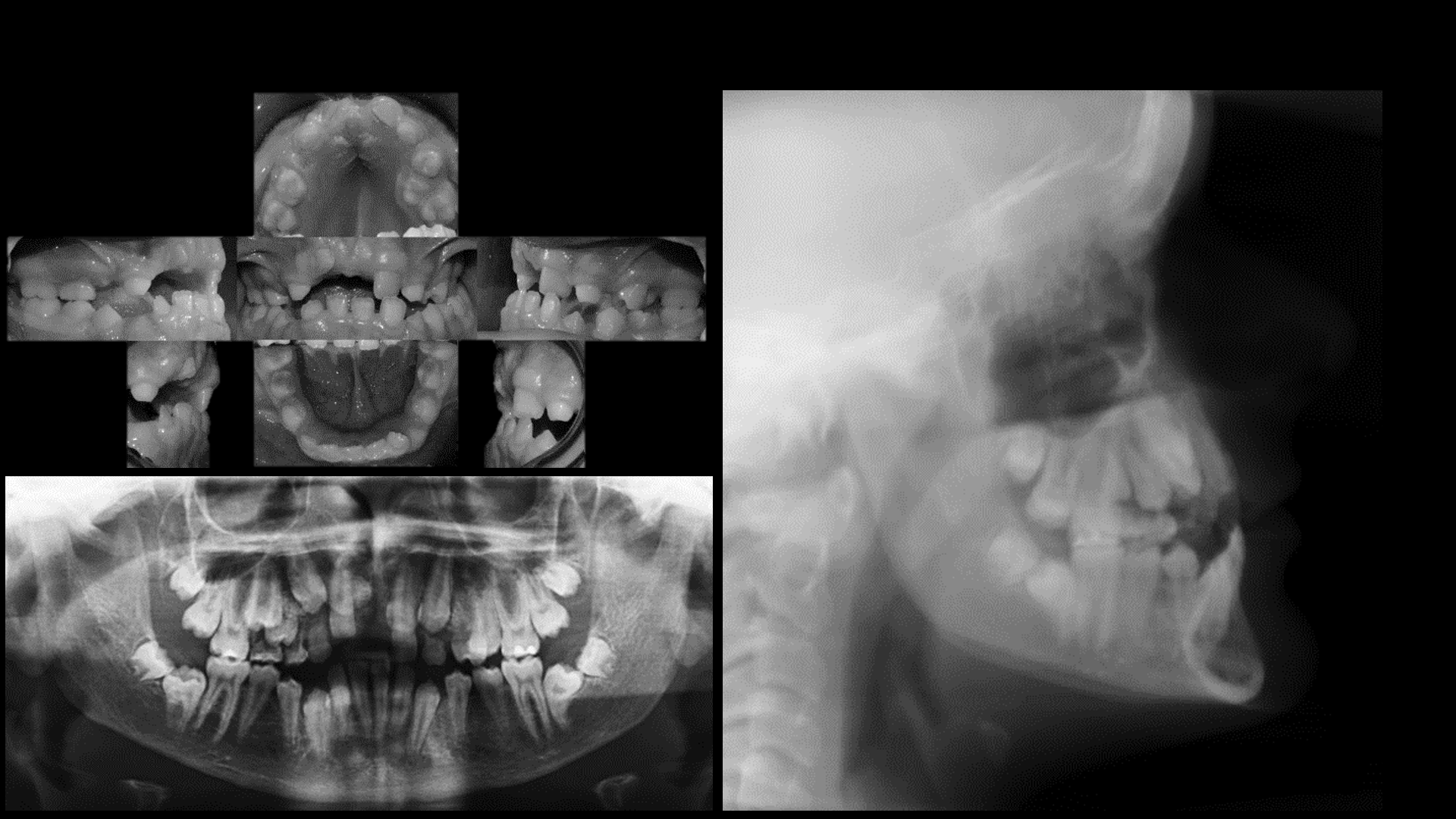


**Appendix figure 3:** CCD case three (fourteen year-old). Intraoral photos, orthopantomography and lateral cephalogram.


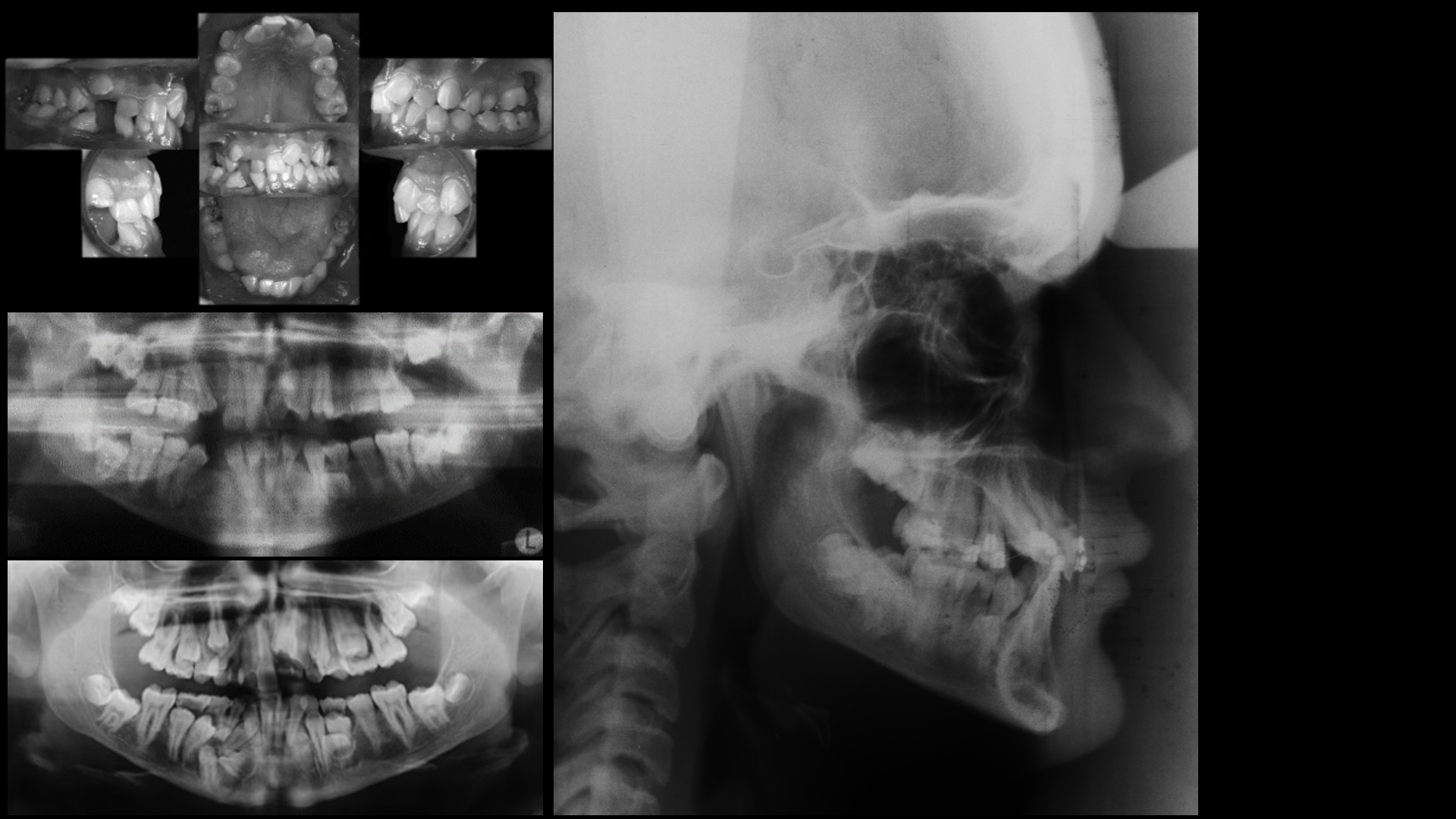


**Appendix figure 4:** CCD case four (sixteen year-old). Intraoral photos, orthopantomography (before and after extraction of the supernumerary teeth) and lateral cephalogram.


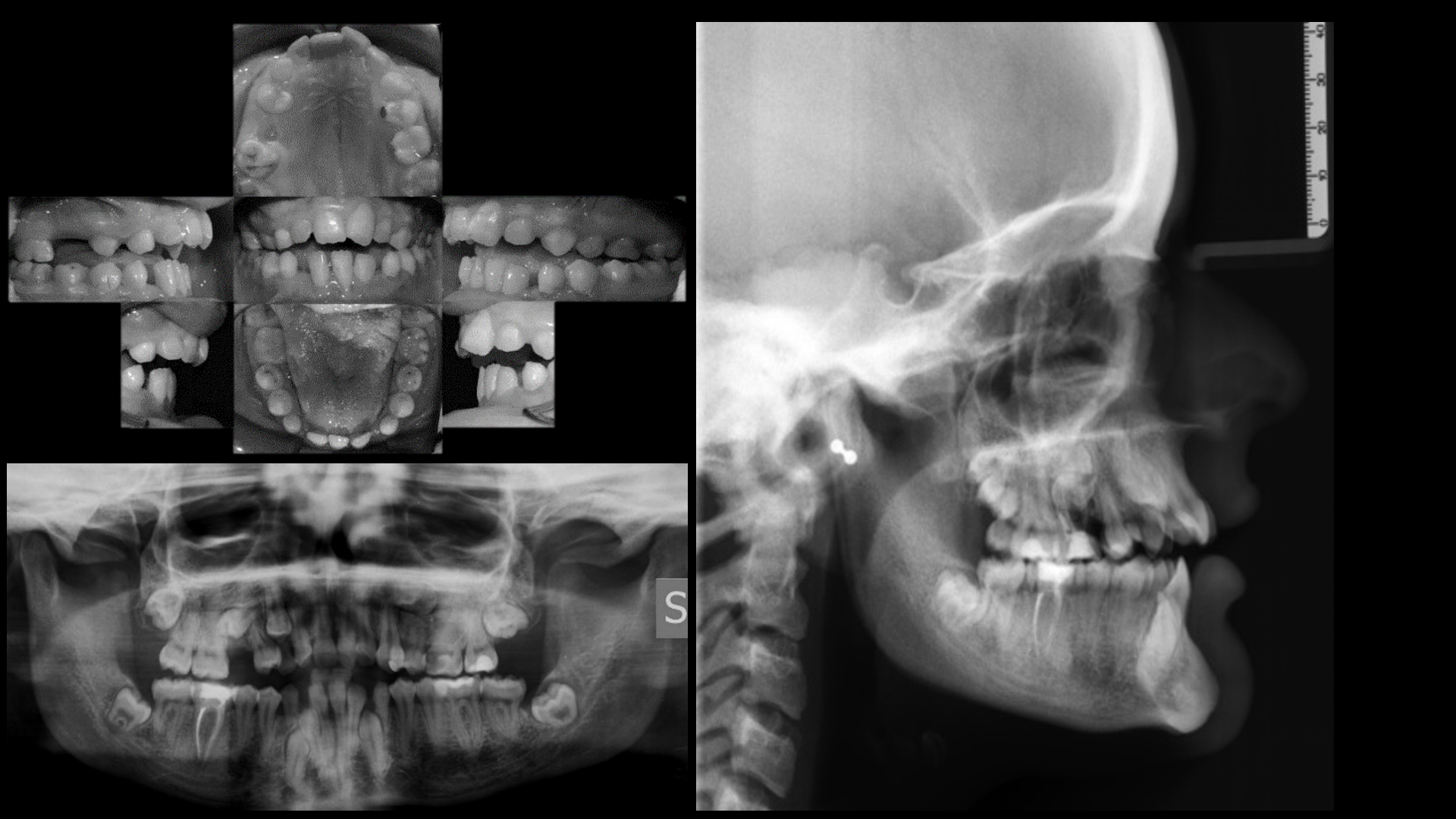


**Appendix figure 5:** CCD case five (twenty-two year-old). Intraoral photos, orthopantomography and lateral cephalogram.
